# Mothers’ Perceptions of the Quality of Postnatal Care Provided in Health Centers and the Associated Factors: A Cross-Sectional Study

**DOI:** 10.30476/IJCBNM.2021.90057.1663

**Published:** 2022-04

**Authors:** Maryam Ahmadinezhad, Fatemeh Vizeshfar, Azadeh Pakniat

**Affiliations:** 1 Mother and Child Welfare Research Center, Hormozgan University of Medical Sciences, Bandar Abbas, Iran; 2 Community Based Psychiatric Care Research Center, Department of Nursing, School of Nursing and Midwifery, Shiraz University of Medical Sciences, Shiraz, Iran; 3 Department of Nursing, School of Nursing and Midwifery, Shiraz University of Medical Sciences, Shiraz, Iran

**Keywords:** Mothers, Mother-child relations, Perception, Postnatal period, Postnatal care

## Abstract

**Background:**

Postnatal care plays a great role in the health of mothers and their neonates. This study aimed to evaluate the mothers’ perceptions of the provided postnatal care and the associated factors.

**Methods:**

In this cross-sectional study, the health centers of Sirik city in Hormozgan province, Iran, were selected using convenience sampling. The study was conducted on 160 mothers who had referred to the selected centers for postnatal care from 7 August 2018 to 2 August 2020 and had given birth to live full-term neonates (>37 weeks of gestation) 40 days to 12 months before sampling. The Mothers’ Perceptions of the Quality of Postnatal care questionnaire was designed by the research team; it included 18 questions about mothers’ perception of care. The collected data were analyzed using the SPSS software, version 21.

**Results:**

The mothers’ mean score of perception was 69.84±16.04; most mothers rated the provided postnatal care and their relationship with the personnel as good or excellent. The mean total scores of the mothers’ perceptions were not different based on their satisfaction with postnatal care (P=0.646) and time of the first referral after birth (P=0.251), but they were significantly different according to the number of referrals (P=0.023) and their satisfaction with the health personnel (P<0.001).

**Conclusion:**

The study results revealed that mothers had a good perception about postnatal care provided by health center staff. Hence, it is necessary to educate all health staff in this regard to provide high-quality postnatal care to all mothers who refer to these centers.

## INTRODUCTION

Pregnancy and childbirth are the most important events in a woman’s life. However, many mothers and/or health staff might neglect the importance of postnatal care. ^1^ Evidence has revealed the insufficiency of the nursing education given to mothers at the time of discharge. ^2^ Nonetheless, it is necessary to provide mothers with sufficient postnatal training, especially about the postpartum warning signs and complications, which can result in reduced maternal mortality and morbidity. ^3 , 4^ It will also exert a positive effect on the mothers’ knowledge and improvement of their self-efficacy. ^5^ The World Health Organization (WHO) has also emphasized the importance of the care provided to mothers during the postpartum period. 

In this period, sufficient training can be provided to mothers about their own as well as their neonates’ health status. ^6^ Mothers, as the primary care providers to their neonates, should be aware of the necessary neonatal care including feeding, hygiene, vaccination, and signs of sickness. It is of great importance to make mothers aware of the necessary care for their own health, as well. Such care includes nipple care, personal hygiene, vaginal hemorrhage, infection, and warning signs, which can be effective in reducing the mothers’ morbidity and mortality. ^7^ Although referral to health centers for receiving postnatal care has been strongly recommended, ^8^ there are several barriers against the timely referral of mothers such as mother’s disabilities and presence of another child at home. ^9^

In addition, most mothers are sleep deprived and may not have sufficient time to refer for postnatal care, especially mothers with an unintended pregnancy and insufficient support and those who did not seek prenatal care. ^10^ Moreover, some mothers may develop psychological disorders such as postpartum depression and psychosis, which cause them to suffer in silence. This will have a negative impact on children’s socio-emotional and cognitive development, as well. ^11^ These problems and their effect on maternal and fetal health indicate the importance of postnatal care.

A review of studies has indicated that the time and number of referrals for postpartum care vary based on cultural, behavioral, and ethnic factors, ^12^ which will in turn reduce their health-related quality of life in the postnatal period. ^13^ Furthermore, the quality of the provided care may not be well enough to fulfill all mothers’ needs, as evaluation of the perspectives of Iranian mothers showed insufficiencies, especially in terms of reliability in the provided postnatal care. ^14^ In this context, the parents’ perceptions of postnatal care have been found to play a pivotal role in the uptake of postnatal care. ^15^ Many studies have shown some reasons for not using postpartum care services in developing countries such as cultural beliefs, parents’ perception of the postpartum period, and postnatal care, and their poor knowledge about maternal morbidity and mortality play a vital role in providing postnatal care. ^16 , 17^ In addition, the outcomes also put forward some significant barriers to postnatal care, which include lack of knowledge of postnatal care, long waiting time for visit, and separation of the mother and baby care in clinics. ^18 , 19^ It is not clear exactly why some women do not use these services despite the provision of postpartum care in health centers. In fact, it all has to do with mothers’ satisfaction and perception of care. ^16 , 19^ The role of the health care system and health care worker factors is very important. Parents complained about lack of attention from health providers and lack of essential services and medical supplies in health facilities. ^16^ Accordingly, it is of utmost importance to monitor the quality of the postnatal care provided by health centers from mothers’ perspectives. The present study aimed to evaluate the mothers’ perceptions of the provided postnatal care at the health centers of Sirik city in Hormozgan province and to determine the factors associated with better perceptions.

## MATERIALS AND METHODS

The present prospective, cross-sectional study was performed at the health centers of Sirik city in Hormozgan province, south of Iran from 7 August 2018 to 2 August 2020. Considering a confidence interval of 95%, a maximum acceptable absolute error of 4, and standard deviation of 23 according to a pilot study and using the following formula

$n=\frac{{\left(Z_{1-\alpha /2}\right)}^{2}S^{2}}{{\left(d\right)}^{2}}$ , 

a 128-subject sample size was determined for the study. At the beginning of the study, it was predicted that some mothers were not willing to participate in the study and some would receive a questionnaire but did not give back or returned incomplete questionnaires, so 25% was considered for sample attrition, and 32 mothers were added to the calculated number for sample. Thus, the sample size increased to 160 subjects. The women who had given birth to live full-term neonates (>37 weeks of gestation) at least 40 days and at most 12 months before sampling were enrolled into the research. At first, they were explained about the study objectives and were asked to sign written informed consent forms. In case the women did not refer to the center routinely, they were excluded and replaced by other individuals. 

The individuals who had referred to the selected centers for receiving postnatal care were selected via convenience sampling. First, three centers from all comprehensive health centers in Sirik city with active mother care programs were selected by cluster sampling. Then, all pregnant mothers who met the inclusion criteria were invited to take part in the research. After explaining the study objectives to the women, we asked them about demographic information, and obstetrics history and wanted them to fill out the Mothers’ Perceptions of the Quality of Postnatal Care questionnaire designed by the research team including18 questions about the mothers’ perception of care. Demographic characteristics included mother’s age, age at marriage, occupation and education level, and husband’s level of education. In addition, obstetric characteristics included the number of pregnancies, interval between pregnancies, delivery method in the last pregnancy, single or multiple births, age at pregnancy, the referral place for prenatal care, neonate’s feeding method, duration of breastfeeding, duration of labor, and the health staff who provided the care. This information was recorded through self-report. Satisfaction scale of this questionnaire was based on the scale designed by Hall et al. for older mothers and ^20^ Dimatteo and Hays to assess the physicians’ satisfaction. ^21^ Because of the nature of the items in these questionnaires and their validity, ^22^ in the present study, the questionnaire was modified and psychometrically designed to have a valid and reliable tool for assessing the mothers’ perceptions of postnatal care provided in comprehensive health centers. The Mothers’ Perceptions of the Quality of Postnatal Care questionnaire consisted of 18 main items including the following sections:

The first section included six questions about the mothers’ psychological support and information they received from midwives, participation in decisions, feeling free to talk with midwives, follow-up of problems, and reception of sufficient training about maternal and neonatal care. In this section, the mothers were asked to respond to each question from 1 indicating ‘not completely’ to 7 indicating ‘very much’. Thus, the total score of this section could range from 6 to 42. In case the visits were handled by another staff rather than the midwife, the mothers were asked to complete the next part, which included the same questions with “the medical staff” instead of “the midwife”. The total score of the mothers’ perception was calculated by the sum of scores.

The second section included 12 statements about the mothers’ satisfaction with the provided care. The mothers were asked to score each sentence from 0 representing ‘completely disagree’ to 5 indicating ‘completely agree’. Hence, the total score of this section ranged from 0 to 60, with higher scores representing the mothers’ higher satisfaction levels. It should be mentioned that the satisfaction scores were reported separately. The total score of Mothers’ Perceptions of the Quality of Postnatal Care between was 6-102. Face, content, and construct validity as well as reliability of this questionnaire have been found to be appropriate. The impact coefficient of all the items was above 1.5, and the questionnaire had appropriate face validity. In addition, the Content Validity Index (CVI) of the questionnaire items ranged from 0.79 to 0.9, which were above the minimum acceptable value, i.e. 0.79. The scale-level index (S-CVI) was also 0.73. Moreover, the Content Validity Ratio (CVR) of the items ranged from 0.62 to 1, while the CVR of two items was less than 0.62. These items were modified and re-evaluated; then, their CVRs were found to reach the acceptable level. Furthermore, the Kaiser-Meyer-Olkin (KMO) index was 0.825 at the first output. Both positive and negative skewness of the data were also within the acceptable range. Thus, the sample size was sufficient for factor analysis. According to Bartlett’s test, explanatory factor analysis was significant for identifying the factor model structure at P<0.001. The Scree Plot based on factor analysis showed a correlation between Mothers’ Perceptions of the Quality of Postnatal Care Questionnaire items. Cronbach’s alpha coefficient for the questionnaire was calculated 0.668. Moreover, Intraclass Correlation Coefficient (ICC) was 0.688, which confirmed its reliability.

Questionnaires were provided to mothers when they referred to the health centers for postpartum visits, and their questions were followed up by phone and their questions answered.

The results were presented as mean±Standard Deviation (SD) for quantitative variables and frequency (percentage) for categorical ones. One-sample Kolmogorov-Smirnov test was used to determine the normal distribution of the data, and Levene’s test was used to test the equality of variances. In this study, the scores of the mothers’ perception were the normal continuous variable and were compared using t-test or analysis of variance (ANOVA) test. All statistical analyses were carried out using IBM SPSS Statistics for Windows, version 21.0 (IBM Corp. 2012. Armonk, NY: IBM Corp.), and P<0.05 was considered statistically significant.

The present study was approved by the Research Ethics Committee of Hormozgan University of Medical Sciences (No. IR.HUMS.REC.1397.118). All participants were required to sign written informed consent forms after receiving verbal explanations about the study objectives and procedures. The participants were also assured about their anonymity as well as the confidentiality of their information.

## RESULTS

This study was conducted on 160 mothers, most of whom (138; 86.2%) were housewives. In addition, the husbands of 124 (77.5%) of the participants were self-employed. Besides, 99 (61.9%) mothers and 83 (51.9%) husbands had academic education. Moreover, the age at marriage was <20 years in about 64 (40%) mothers (Table 1).

**Table 1:** The mean scores of the mothers’ perceptions of postnatal care according to their demographic characteristics

Variable	Categories	No. (%)	Mothers’ psychological support		Mothers’ satisfaction		Total score of mothers’ perceptions	
			Mean±SD	P value	Mean±SD	P value	Mean±SD	P value
Age	<30 years	94 (58.8)	27.40±14.34	0.280*	40.81±5.49	0.146*	68.22±15.60	0.128*
	>30 years	66 (41.3)	29.89±14.22		42.25±6.95		72.15±16.49	
Mother’s occupation	Homemaker	138 (86.2)	28.01±14.29	0.358*	41.73±6.24	0.101*	69.74±16.05	0.848*
	Employee	22 (13.8)	31.04±14.37		39.40±5.27		70.45±16.35	
Spouse’s occupation	Self-employed	124 (77.5)	27.77± 14.38	0.642*	40.98±6.26	0.111*	68.75±16.49	0.304*
	Employee	36 (22.5)	29.05±14.37		42.88±6.14		71.94±15.16	
Mother’s education level	Up to high school	61 (38.1)	30.13±13.81	0.239*	42.16±6.13	0.227*	72.29±15.32	0.130*
	Academic	99 (61.9)	27.38±14.56		40.94±6.15		68.33±16.36	
Spouse’s education level	Up to high school	77 (48.1)	29.05±14.37	0.599*	41.77±6.37	0.470*	70.83±16.57	0.455*
	Academic	83 (51.9)	27.85±14.58		41.07±5.96		68.92±15.58	
Age at marriage	<20 years	64 (40)	29.78±14.18	0.574**	41.68±4.01	0.572**	71.46±15.05	0.444**
	20-25 years	63 (39.4)	27.09±13.73		40.79±6.78		67.86±16.09	
	>25 years	33 (20.6)	28.36±14.30		42.06±8.14		70.41±17.90	

*Independent *t*-test; **One way ANOVA

The mean score of the mothers’ perception was 69.84±16.04 (Rang: 27-102). According to Table 1, the mean scores were not different based on demographic characteristics (P>0.05). The differences in the mean scores of the mothers’ perceptions according to obstetric and neonatal feeding characteristics are presented in Table 2. They were different according to the mothers’ number of pregnancies (P=0.041) and the medical staff in charge (P=0.032). The mean score of mother’s satisfaction was significantly different in the gestational age group (P=0.025). Table 3 shows the mean scores of the mothers’ perceptions of postnatal care according to their satisfaction with the provided care, satisfaction with the personnel, and time and number of postnatal referrals. According to the summarized data in this Table, most of the mothers (108; 67.5%) rated the provided postnatal care as good, 27 (16.9%) as excellent, and the others as moderate to weak. 130 mothers (81.25%) referred to the health center sooner than one week after delivery and 86 (53.75%) referred <5 times during the interval between delivery and the beginning of the study. Moreover, most of the mothers 148 (92.5%) rated their relationship with the personnel as good or excellent. According to the results presented in Table 3, the total mean scores of the mothers’ perceptions were not different according to the mothers’ satisfaction with postnatal care and the time of the first referral after delivery (P>0.05), but they were significantly different based on their satisfaction with the health personnel (P<0.001) and number of referral (P=0.023).

**Table 2:** The mean scores of the mothers’ perceptions of postnatal care according to their obstetric and neonatal feeding characteristics

Variable	Categories	No. (%)	Mothers’ psychological support		Mothers’ satisfaction		Total score of mothers’ perceptions	
			Mean±SD	P value	Mean±SD	P value	Mean±SD	P value
Number of pregnancies	0-1	59 (36.90)	26.18±15.35	0.130*	40.27±6.04	0.073*	66.45±16.70	0.041*
	≥2	101 (63.10)	29.74±13.55		42.07±6.15	71.82±15.38		
Interval between pregnancies (year)	<2	36 (22. 60)	29.38±13.93	0.947**	40.44±5.24	0.636**	69.83±17.08	0.636**
	2-5	75 (46.70)	29.91±13.36		41.96±5.09		71.87±14.32	
	>5	49 (30.70)	30.66±14.06		42.03±8.45		72.70±17.24	
Previous delivery method	Natural vaginal delivery	98 (61.30)	27.40±14.61	0.281*	41.28±6.18	0.776*	68.69±15.58	0.284*
	Cesarean section	62 (38.70)	29.93±13.83		41.57±6.21		71.50±16.81	
Single and multiple pregnancy	Singleton	153 (95.60)	28.68±14.20	0.289*	41.52±6.22	0.432*	70.20±15.98	0.212*
	Twins or triplets	7 (4.40)	22.33±18.02		39.50±4.25		61.83±18.08	
Gestational age (week)	<37	19 (11.90)	22.73±12.86	0.064*	44.36±5.23	0.025*	67.10±15.74	0.430*
	>37	141 (88.10)	29.19±14.35		41.01±6.18		70.21±16.10	
Type of care center	Health center	114 (71.40)	28.26±14.32	0.787*	41.34±5.85	0.729*	69.61±15.70	0.707*
	OB private clinic	46 (28.60)	27.42±15.08		40.88±7.78		68.30±18.12	
Type of feeding	Breastfeeding	144 (90)	28.42±14.30	0.984*	41.51±6.22	0.534*	69.93±16.17	0.825*
	Formula	16 (10)	28.50±14.77		40.50±5.59		69.00±15.34	
The medical staff in charge	Midwife always	51 (31.90)	29.48±12.38	0.001**	40.55±6.65	0.084**	70.04±14.54	0.032**
	Other trained personnel	92 (57.70)	30.46±14.32		41.27±6.14		71.73±16.80	
	different in each visit	17 (10.40)	18.08±14.23		43.95±4.92		62.04±14.79	
Duration of breastfeeding (months)	<3	24 (15)	27.16±13.68	0.588**	42.05±5.84	0.726**	69.22±14.77	0.810**
	3-6	21 (13.12)	24.75±13.44		42.50±8.79		67.25±16.88	
	6-9	13 (8.12)	25.25±16.65		40.62±5.92		65.78±19.73	
	≥12	102 (63.75)	29.33±14.51		40.98±5.34		70.32±16.00	

*Independent *t*-test; **One-way ANOVA

**Table 3:** The mean scores of the mothers’ perceptions of postnatal care according to their satisfaction with the provided care, satisfaction with the personnel, and time and number of postnatal referrals

Variable	Categories	No (%)	Mothers’ psychological support		Mothers’ satisfaction		Total score of mothers’ perceptions	
			Mean±SD	P value	Mean±SD	P value	Mean±SD	P value
Satisfaction with the provided postnatal care	Moderate to weak	25 (15.6)	25.88±12.58	0.600**	41.24±7.42	0.960**	67.12±14.09	0.646**
	Good	108 (67.5)	28.72±14.75		41.50±6.35		70.23±16.87	
	Excellent	27 (16.9)	29.62±14.15		41.18±3.83		70.81±14.53	
Satisfaction with the personnel	Moderate to weak	12 (7.5)	13.00±10.86	<0.001**	39.00±6.03	0.290**	52.00±14.33	<0.001**
	Good	75 (46.9)	27.80±13.72		41.96±6.71		69.76±15.40	
	Excellent	73 (45.6)	31.61±13.78		41.24±5.52		72.86±15.19	
Time of first referral after childbirth (day)	1-7	130 (81.25)	28.11±14.43	0.322*	40.60±5.26	0.464*	68.70±15.53	0.251*
	>7 days	30 (18.75)	32.45±10.80		41.81±4.85		74.27±12.37	
Number of referrals	<5	86 (53.75)	32.73±12.02	0.582**	42.34±5.56	0.137**	75.08±13.32	0.023**
	5-10	52 (32.5)	27.64±15.85		40.07±5.64		67.71±16.94	
	>10	22 (13.75)	25.50±15.06		40.60±9.64		66.10±19.62	

*Independent *t*-test; **One-way ANOVA

## DISCUSSION

The results of the present study indicated the mothers’ satisfactory perceptions of the provided postnatal care at the health centers. It should be noted that the questionnaire used in this study covered important aspects of postnatal care including information, support, and relationship with the medical staff. The results of this study indicated that most of the mothers had a positive perception about postpartum care; most of them described their relationship with personnel as good or excellent, but studies in the world have shown different results in maternal satisfaction from different dimensions of postpartum care. An evaluation of mothers in Australia demonstrated that most of the mothers were happy with most of the postnatal care provided in relation to baby care and mothers’ recovery, but lower scores were reported with respect to the midwives’ availability; also, maternal health needs were different with our study. ^23^ The reason for this difference can be the difference in culture and structure of the health system .In another study, the subjective importance of all postnatal care variables was scored higher than the perceived reality for all statements, which indicated that the mothers were not satisfied with the postnatal care. ^24^ It should be mentioned that the major determinant of mothers’ perspectives and perceptions of postnatal care is the quality of the care provided at health centers, which justifies the controversy among the results. Another study assessed the perception of women who referred to health centers for receiving postnatal care. The results showed a negative perception about postnatal care, ^25^ which was not in the same line with the results of the present study. It is noteworthy that the assessment tool used in the above-mentioned study differed from that utilized in the current investigation. It should also be noted that the above-mentioned studies followed a qualitative approach, while the answers were categorized based on a Likert scale and scored in the present research in order to obtain more accurate results.

In the current study, the demographic characteristics of the mothers and their husbands such as age, education level, and occupation did not affect the mothers’ perception scores. However, the mothers’ number of pregnancies and the medical staff in charge had a statistically significant relationship with the mothers’ perceptions. Research results are highly variable in relation to the demographic and other characteristics of mothers and perception about postnatal care. One study reported that the mothers’ age, education level, and number of children were not associated with the quality gap of postnatal care. ^17^ Another study indicated that the mothers’ perceptions of the quality of postnatal care were not affected by their age, but were associated with their education level. ^26^ Some studies expressed the relationship between the mothers’ satisfaction and method of feeding, age, parity, and gravidity. ^25 , 27 , 28^ The discrepancy between the results could be attributed to different categorizations of the education levels as well as to the confounding effects of cultural and behavioral factors on the results.

As to obstetric characteristics, parity has been mentioned as an important factor in the perception of postnatal care, and first-time mothers have been reported to have a lower perception. ^13^ This was confirmed by the results of the present study. Another study also disclosed that first-time mothers had a worse perception of postnatal care compared to multiparous women. ^29^ Several studies have thus focused merely on the perceptions of first-time mothers. ^30 , 31^ The results of these studies are different, which may be due to differences in the study environment or data collection tools. In contrast, the findings of another research revealed no significant difference in the mothers’ perceptions of the quality of postnatal care according to parity and gravidity. ^25^ These controversial results might be associated with different information sources and social supports among mothers under investigation. 

Another significant determinant of the mothers’ perceptions of postnatal care was the care provider in the current research. Based on the findings, the mothers visited by midwives had lower perception scores compared to those who received care from other trained personnel. Another research also referred to poor communication and clinical skills in midwives, which were associated with the mothers’ low satisfaction with the quality of postnatal care. ^31^ These results were consistent with those of the present study. On the contrary, another study indicated the higher perception of mothers in relation to the postnatal care provided by midwives. ^25^ The present study findings could result from the midwives’ workload, which reduced the quality of their care provision. These results emphasized the necessity to devise a standard postnatal program at these centers and to educate healthcare providers in order to provide all mothers with high-quality postnatal care. ^32^

In the present study, most mothers rated the provided postnatal care and their relationship with the personnel as good. Similarly, another study in the city of Mashhad demonstrated that most mothers were satisfied with the provided postnatal care including consultation, training, technical expertise, and interpersonal communication. ^31^ Moreover, the results of the present study showed that satisfaction with the health personnel was an important determinant of the mothers’ perception of postnatal care. Other studies have also revealed the relationship between women and care providers as one of the important factors affecting the mothers’ satisfaction with postnatal care. ^26 , 33^ In the same line, another study demonstrated that higher rates of communication with health professionals were associated with first-time mothers’ higher satisfaction with postnatal care. ^34^ Another finding of this study was a statistically significant relationship between the number of referrals and mothers’ perceptions of care. Findings from other studies highlight the importance of the maternal referral system. ^35 , 36^ These results indicated the importance of communication and relationship with the mother during postnatal visits.

One of the limitations of the present study was its cross-sectional design, which limited the evaluation of follow-up results and assessment of the causal relationships between the variables. Additionally, the study outcomes could be affected by several confounding factors such as mother’s psychological status, social support, economic status, and personal beliefs. Furthermore, since the participants were selected from one province, the results cannot be generalized to the whole country due to the differences in economic and ethnic backgrounds.

## CONCLUSION

The study results revealed that the majority of the mothers had positive perceptions of postpartum care in the health centers. It is necessary to maintain and promote the mothers’ satisfaction and ensure the quality of care. In order to achieve this goal, it is necessary for health planners to complete this questionnaire as a part of the mother care program. It is also essential to pay greater attention to the postnatal care provided at the studied health centers, devise a standard guideline, and educate all health staff to provide high-quality postnatal care to all mothers who refer to these centers. These guidelines are recommended to include the aspects highlighted in this study, which can help the policymakers plan a more efficient strategy.

## ACKNOWLEDGEMENT

The authors would like to acknowledge the Research Deputy and Research Committee of Mother and Child Welfare Research Center, Hormozgan University of Medical Sciences for their support (No. 960113). They would also like to thank the personnel of Health and Treatment Center of Sirik, Hormozgan province, Iran for their cooperation. Ms. Roghayyeh Malahi and Ms. Maryam Atin are also appreciated for making the necessary arrangements with the health centers as well as for providing access to the samples. Thanks also go to Ms. A. Keivanshekouh at the Research Consultation Center (RCC) of Shiraz University of Medical Sciences for her invaluable assistance in improving the use of English in the manuscript. The authors of the present study declare that the study was financially supported by the Research Deputy of Hormozgan University of Medical Sciences.

**Conflict of Interest:** None is declared. 
